# Construction of power network security risk assessment model based on LSA-SVM algorithm in the background of smart grid

**DOI:** 10.1038/s41598-024-59473-x

**Published:** 2024-04-20

**Authors:** Haojin Qi, Wan Zhu, Mingda Ye, Yichen Hu, Yong Wang

**Affiliations:** State Grid Zhejiang Electric Power Co., Ltd. Ningbo Power Supply Company, Ningbo, 315000 China

**Keywords:** Support vector machine, Smart grid, Flash search algorithm, Power network, Security risk evaluation model, Computer science, Information technology, Software

## Abstract

Due to theintricate and interdependent nature of the smart grid, it has encountered an increasing number of security threats in recent years. Currently, conventional security measures such as firewalls, intrusion detection, and malicious detection technologies offer specific protection based on their unique perspectives. However, as the types and concealment of attacksincrease, these measures struggle to detect them promptly and respond accordingly. In order to meet the social demand for the accuracy and computation speed of the power network security risk evaluation model, the study develops a fusion power network security risk evaluation algorithm by fusing the flash search algorithm with the support vector machine. This algorithm is then used as the foundation for building an improved power network security risk evaluation model based on the fusion algorithm.The study's improved algorithm's accuracy is 96.2%, which is higher than the accuracy of the other comparative algorithms; its error rate is 3.8%, which is lower than the error rate of the other comparative algorithms; and its loss function curve convergence is quicker than that of the other algorithms.The risk evaluation model's accuracy is 97.8%, which is higher than the accuracy of other comparative models; the error rate is 1.9%, which is lower than the error rate of other comparative models; the computing time of the improved power network security risk evaluation model is 4.4 s, which is lower than the computing time of other comparative models; and its expert score is high. These findings are supported by empirical analysis of the improved power network security risk evaluation model proposed in the study. According to the study's findings, the fusion algorithm and the upgraded power network security risk evaluation model outperform other approaches in terms of accuracy and processing speed. This allows the study's maintenance staff to better meet the needs of the community by assisting them in identifying potential security hazards early on and taking the necessary preventative and remedial action to ensure the power system's continued safe operation.

## Introduction

Given the society's ongoing progress and the economy's exponential growth, the reliability and reliability of the power supply are critical for the smooth operation of modern society. The power network (PN) is, however, increasingly exposed to major security concerns as a result.Due to its complexity and size, the PN is vulnerable to interference and attacks from both internal and external forces, which can cause the power system (PS) to become unstable and paralysed^1^. PSs must promptly and accurately assess and identify any potential security concerns in order to guarantee the safe operation of PNs. However, due to their lengthy assessment cycles and unreliable outcomes, existing PN security evaluation approaches are frequently unable to satisfy the demands of the PN environment, which is changing quickly^2^. The Support Vector Machine (SVM), a prominent machine learning algorithm, classifies samples by constructing a high-dimensional feature space, mapping the samples into that space, and then selecting the optimal hyperplane there^3^. The SVM method may create a classification model for estimating the likelihood of occurrence of potential hazards in PS by learning the characteristics and classification labels of the sample data in PN security risk evaluation. The SVM algorithm's configurable parameters have a significant impact on its accuracy and robust performance, though, and it has a lengthy training cycle^4^. It is advisable to consider the selection of the parameters using the Lightning Search technique (LSA) technique in order to swiftly choose the best SVM parameters.By modeling the formation and propagation of lightning, the LSA method is able to locate the global best solution in the search space^5,6^. Because of this, the study suggests combining the LSA algorithm and SVM algorithm to create the LSA-SVMPN security risk evaluation algorithm. This algorithm forms the basis for creating the Power Network Security Risk Evaluation Model (PNSREM), which utilizes the LSA-SVM algorithm and is anticipated to enhance the security and dependability of PSs through auxiliary support. The theoretical contribution of the work is that PNSREM fully exploits the benefits of the LSA and SVM algorithms to improve the efficacy and accuracy of the assessment model. The practical application's contribution is to give PS managers a basis for making decisions that will help them run and manage PSs more efficiently. In Section “Introduction”, the study discusses how SVM, LSA, and PS security assessment techniques are currently progressing. The LSA-SVM algorithm, the PCNSecurity Risk Evaluation Model (SREM), and the Security Risk Assessment Indicator System (SRAIS) are built in Section “Related works”. The proposed LSA-SVM method and PNSREM are empirically investigated in part III of the research. Section “Empirical analysis of PNSREM based on LSA-SVM algorithm” provides the conclusion and prediction for future research directions.

## Related works

With the rapid advancement of artificial intelligence, optimisation and machine learning algorithms have a wide range of applications in a variety of fields. In order to minimise the power loss in the distribution system, Chinnaraj et al. proposed to construct a renewable distribution and generation coefficient calculation model by using the LSA modified complex method, and studied the effectiveness of the model, which was found to redistribute the distribution and generation coefficients of the distribution system^7^. Robert et al. proposed combining quantum behaviour with LSA to construct a multi-objective hybrid computational model to solve the problem of economic emission scheduling of different energy generating units, and simulation experiments were carried out on the model, and it was found that the model had better computational and scheduling performance than other traditional models^8^. Neffati et al. proposed to construct a three-dimensional brain magnetic resonance image classification model using SVM, and validated the effectiveness of the model, and found that the model has better classification performance and robustness than other comparative models, and can assist medical staff in diagnosing the medical images^9^. In addition, for security detection in other fields, Yazdinejad et al. proposed using machine learning technology and federated learning models to construct privacy protection models for healthcare electronic products. They used auditing mechanisms to update and identify data, thereby improving the performance of the model and reducing the risk of privacy leakage^10^. Namakshenas et al. proposed using federated learning techniques and quantum strategies to train datasets to address the issue of consumer privacy protection in the Internet of Things, thereby improving the accuracy of dataset detection and ensuring consumer privacy security^11^. Yazdinejad et al. proposed using blockchain technology to protect and detect abnormal data in the Internet of Things, thereby improving the detection accuracy of the search model^12^. Yazdinejad et al. proposed using federated learning methods and synchronous asynchronous methods to construct a hybrid privacy protection federated model in the next generation of IoT hybrid privacy protection. Through performance evaluation of quality data, the accuracy of the model in data detection was demonstrated, and the privacy security of the system was ensured^13^. The above scholars have demonstrated high detection performance advantages in the application of blockchain technology in the Internet of Things and its privacy protection, thereby protecting the security and privacy of related data. However, federated learning technology and blockchain technology still pose a risk of privacy leakage when computing and trading data. Therefore, in future research, it is necessary to use encryption algorithms or consensus mechanisms to increase the security of data privacy. The LSA algorithm used in the study has made technical improvements to SVM, while taking into account the data situation of power grid security risk assessment, thereby enhancing the advantages of data privacy protection.

As the research of machine learning algorithms is further deepened, there are more and more researches applying them to PN. To improve the accuracy and computational performance of the PS fault prediction model, Li et al. proposed building a complex PS fault prediction model based on genetic algorithms and SVM, and analysing its effectiveness. They discovered that the model's prediction accuracy is 98.87%, and the identification accuracy of the fault region is 94.91%, which has practical application value^14^. Aiming at the problem of high training cost of traditional PS security assessment model, Thams et al. proposed an improved and highly scalable algorithm based on complex network theory, and empirically analysed the algorithm, and found that the algorithm's training cost is much lower, and its training time is 10% of that of the traditional algorithm, which is of practical application value^15^. In order to protect PN data security, Chen et al. proposed an improved encryption system architecture based on high-speed encryption chip, and empirically tested the scheme, and found that the scheme can meet the performance of most cryptographic operations, with a certain degree of ubiquity, and has the potential for practical application^16^. In order to conduct real-time analysis of complex PS and respond to emergencies, Silva et al. proposed and risk response only system based on static security analysis, and empirical analysis of the system found that the system can cut off emergencies, and its response accuracy rate is close to 59%, which is of practical use value^17^. In order to improve the stability of the short-term voltage of the power system, Li et al. used a new deep learning intelligent system combined with data enhancement, and introduced a generative adversarial network to expand the data set, and then established an evaluation model of two-way gating recursive unit and attention mechanism. High classification accuracy and response efficiency were reflected in the dataset test^18^. To improve the security threat of the Internet of Things, Yazdinejad and others designed a security intelligent fuzzy blockchain framework, which uses a novel fuzzy score model, optimized attack detection, fuzzy matching and fuzzy control system to detect the future attacks of the Internet of Things. Finally, the good detection performance of its framework for security threats is obtained and verified by evaluation^19^. About network threat search malicious attack detection, Rabieinejad proposed a in the Etheric fang block chain using a generated against network and deep recursive neural network, to build the network threat search model, and in the model evaluation results of malicious attack detection accuracy as high as 99.98%, to prove the accuracy of the model of network attack search^20^. Regarding the security of industrial control systems, Nakhodchi et al. proposed an application-layer attack detection and attribution model SteeEye model based on semi-deep learning to detect network attacks of industrial control systems and use classification enhancer as the basic predictor of attack attribution. The final experimental results demonstrate the superiority of its model for cyber detection and attribution of the system^21^.

In summary, with more and more research on optimisation algorithms and machine learning algorithms, the LSA algorithm and SVM algorithm have great application prospects in a variety of fields. To satisfy the present-dayneed for accurate power network security risk assessment, the LSA-SVM algorithm is developed through the fusionof the LSA and SVM algorithms. Subsequently, the power network security risk assessment model is generated using this algorithm. Previous studies classified the SVM models to improve the operation efficiency of the corresponding models. Machine learning algorithms are used to build security evaluation models, and then combine encryption systems and other technologies to increase the security of network data. However, the LSA-SVM algorithm developed, on the one hand, to provide pattern feature language for the evaluation model, which is realized by reducing the semantic space. On the other hand, combining the classification performance of the SVM model to reduce the complexity of data calculation and improve the accuracy of risk assessment. LSA has the capability to convert high dimensional characteristics into a semantic space representation of lower dimensions. This is achieved by decreasing the construction of the semantic space model to reduce the complexity and computational overhead of the SVM model. As a result, performance and efficiency can be improved. Furthermore, LSA can assist with uncovering the hidden correlation patterns and characteristics within power networks, thereby enhancing the accuracy of risk assessment. Therefore, the proposed LSA-SVMmodel for evaluating the security risks of power networks can enhance the effectiveness and dependability of power network security risk evaluation.

## Construction of PNSREM based on LSA-SVM algorithm

SVM finds extensive use in pattern recognition, data classification, and regression analysis. Nonetheless, SVM's performance is somewhat reliant on parameters, necessitating an improvement to enhance its operational and risk-detection capabilities. This study thus proposes to enhance SVM using LSA. Additionally, a power network security risk evaluation model is developed, grounded in LSA-SVM, to assess and forecast power system security risk. The work combines the LSA algorithm and the SVM algorithm to increase the accuracy and computing speed of the standard PNSREM, and therefore creates the improved PNSREM for the assessment of PN security risk. Firstly, the study constructs the PN security risk indicator system, and then uses the LSA algorithm to improve the SVM algorithm. Then, the risk is classified and predicted by the LSA-SVM algorithm, so as to construct the PNSREM based on the LSA-SVM algorithm.

### SRAIS construction of PCN

**Figure 1:**
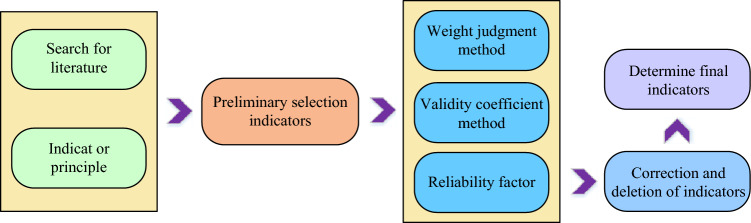
Construction steps of electric power network security risk assessment index system.

PCN is a key infrastructure in PSs, which is responsible for realising the communication and data transmission functions of PSs, and is crucial for the operation and management of PSs. However, as information technology advances and PCN intelligence improves, PCNs are exposed to an increasing number of security threats^22,23^. As a result, it is critical to develop a scientific and acceptable SRAIS for PCNs in order to assure the safe operation of PCNs. To begin, defining the characteristics and security requirements of PCN is the foundation for developing security risk assessment indices. As an important part of PS, the main task of PCN is to transmit and manage various information in PS. Therefore, SRAIS should be considered in terms of scientific, systematic, generic, practical and independent, and the SRAIS of PCN is constructed with this principle. The construction steps of this system are shown in Fig. 1.

In Fig. 1, the experiment will select preliminary PN security risk evaluation indicators based on a large amount of existing literature and the principles of indicator construction^24,25^. Due to the huge amount of information, to ensure the comprehensiveness of the evaluation indicators, the preliminary selection of evaluation indicators is prone to the problem of huge number, which in turn leads to the distortion of the evaluation results. Therefore, the study will use the weighted judgement method to screen these indicators. The weight judgement method is a commonly used method to determine the importance of each indicator in the whole indicator system by assigning weights to it. The study will rely on expert ratings as the basis of weights, and determine the weight value of each indicator through the experience and knowledge of the experts. The calculation equation of the weight judgement method is shown in Eq. (1).

1 $$ f_{i} = \left\{ {\begin{array}{*{20}l} {\lambda_{i} > \lambda_{ch} ,} \hfill & {Sieve \, extraction} \hfill \\ {\lambda_{i} \ge \lambda_{ch} ,} \hfill & {Sieve \, off} \hfill \\ \end{array} } \right. $$

In Eq. (1), $f_{i}$ denotes the $i$th evaluation indicator; $\lambda_{i}$ denotes the weight of $f_{i}$; and $\lambda_{ch}$ denotes the screening weight. Subsequently, the study verified the validity of the screened indicators based on the expert scores to ensure that the screened indicators objectively reflect the security risk of the PN. In the validity validation process, the study uses the validity coefficient to assess the correlation between the screened indicators and the security risk of the PN. The equation for calculating the validity coefficient is shown in Eq. (2).

2 $$ \beta_{i} = \sum\limits_{j = 1}^{n} {\left| {\overline{{x_{i} }} - x_{ij} } \right|} /S*M $$

In Eq. (2), $\beta_{i}$ denotes the validity coefficient of $f_{i}$; $\overline{{x_{i} }}$ denotes the average of the expert ratings of $f_{i}$; $x_{ij}$ denotes the rating of $f_{i}$ by the $j$th expert; $S$ denotes the number of experts; and $M$ denotes the optimal value of the rating. By calculating the validity coefficients, the study was able to determine the predictive ability of each risk assessment indicator for assessing the security risk of PN. In addition to this, the study also assessed the reliability and consistency of the screened indicators through the reliability coefficient. The study calculates the reliability coefficient through multiple assessments and the participation of different experts to ensure that the screened indicators can maintain stable assessment results under different circumstances. Equation (3) shows how to compute the dependability coefficient.

3 $$ \rho_{i} = \sum\limits_{j = 1}^{n} {\frac{{\sum\limits_{i = 1}^{n} {(x_{ij} - \overline{{x_{j} }} )} (y_{i} - \overline{y} )}}{{\sqrt {\sum\limits_{i = 1}^{n} {(x_{ij} - \overline{{x_{j} }} )^{2} \sum\limits_{i = 1}^{n} {(y_{i} - \overline{y} )^{2} } } } }}} /S $$

In Eq. (3), $y_{i}$ represents the average data of the expert ratings of the $S$ times of calculation; the overall mean of the $\overline{y}$ average data. Through the validation of validity and reliability, the study can make timely amendments and deletions to the indicator system to ensure the validity and reliability of the PN security risk evaluation indicator system. Only a strictly screened and verified indicator system can more accurately assess the security risk of PN, provide scientific and reliable decision-making basis for relevant departments, and guarantee the stable operation of PN. Table 1 depicts the PN security risk evaluation index system developed by the study.

**Table 1:** Power network security risk evaluation index system.

Dimension	Code	Index
Network topology	P1	Reliability of the network topology
	P2	The complexity of the network topology
	P3	Fault-tolerance of the network topology
Network access control	P4	Improvement of the access control strategy
	P5	The effectiveness of account management and authority control
	P6	Ability to prevent unauthorized access
Identity authentication evaluation	P7	Reliability of the identity authentication mechanism
	P8	The ability to prevent identity forgery and fraudulent use
	P9	Ability to prevent unauthorized access
Data transfer encryption evaluation	P10	The degree of encryption of the data being transmitted
	P11	Security of the encryption algorithm
	P12	The ability to prevent data leakage and tampering
Data backup and recovery	P13	Integrity of the data backup and recovery mechanism
	P14	Reliability of the data backup and recovery mechanism
Security vulnerability assessment	P15	The existence or absence of security vulnerabilities
	P16	Weak password and not updated patch patches
	P17	Security vulnerability repair and vulnerability management situation

As can be seen from Table 1, the study looks at six dimensions of PNs: topology, access control function, authentication function, security vulnerability, data backup and recovery, and data transmission encryption. Quantitative analysis and qualitative assessment methods can be used and combined with the actual situation for a comprehensive assessment. Such an assessment helps to equationte appropriate security measures and contingency plans to improve the security and reliability of PNs. According to the key facilities of the power system and the method of the power grid security risk evaluation index, the power grid security risk evaluation system is developed and designed, which provides the index basis for the security risk evaluation algorithm.

### construction of PN security risk evaluation algorithm

**Figure 2:**
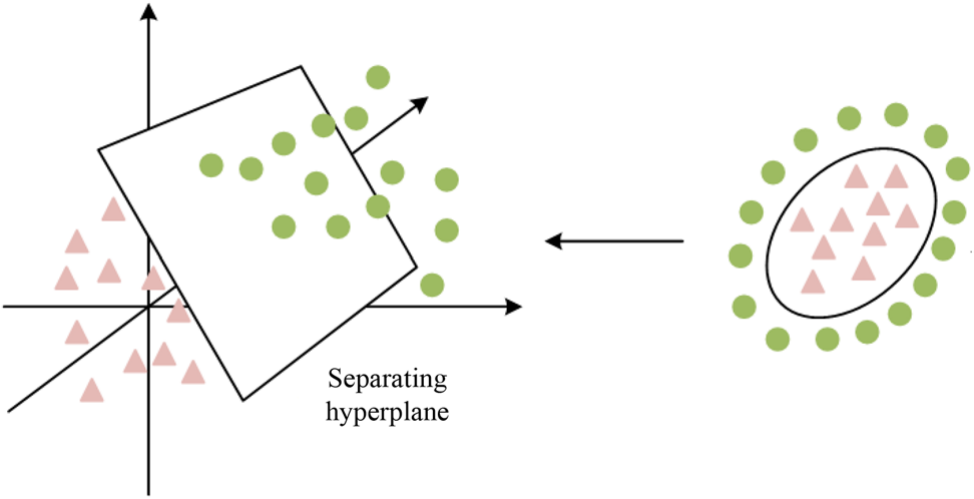
The SVM vector mapping principle.

SVM is a commonly used machine learning algorithm for performing classification and regression tasks, with good handling of high-dimensional feature spaces and small-sample data, as well as non-linear problems^26,27^.The support vector machine (SVM) offers robustness, adaptability and high efficiency when tackling nonlinear issues, making it commonly applied in assessing security risks in network systems. However, SVM has limitations; the abundance of data in power networks can result in high calculations costs if solely usedfor risk assessment. Furthermore, the effectiveness of Support Vector Machines (SVM) in power network security risk evaluation heavily relies on the careful selection of parameters. Thus, it is crucial to enhance the performance of SVM by utilizing optimization algorithms, which can boost accuracy and reliability. SVM operatesby converting low-dimensional space data into high-dimensional space through vector mapping.The principle of SVM vector mapping is shown in Fig. 2.

SVM maps the samples into the high-dimensional feature space, as illustrated in Fig. 2, so that the samples are divided by feature space hyperplanes, as given in Eq. (4).

4 $$ f(x) = b + \omega \varphi (x) $$

In Eq. (4), $\omega$ denotes the weights in the high-dimensional space; $\varphi ()$ denotes the mapping process; $x$ denotes the input sample vector; and $b$ denotes the regression bias item. The equation of the risk function is shown in Eq. (5).

5 $$ R(\omega ) = \frac{1}{2}\left\| \omega \right\|^{2} + C\sum\limits_{i = 1}^{n} {l( - y_{i} + f(x_{i} ))} $$

In Eq. (5), $C$ denotes the penalty factor; $y_{i}$ denotes the mapping vector; $l()$ denotes the high-dimensional mapping. In order to minimise the structural risk, it is investigated to control the accuracy of SVM by linear non-sensitive mapping, which is calculated as shown in Eq. (6).

6 $$ \max (0,\left| { - y_{i} + f(x_{i} )} \right| - \varepsilon ) = l(f(x_{i} ) - y_{i} ) $$

In Eq. (6), $\varepsilon$ denotes the insensitive parameter. The study transforms the regression problem into a dyadic problem by adding operators to the function and based on the Lagrangian dyadic theory, which is shown in Eq. (7).

7 $$ \left\{ {\begin{array}{*{20}c} {\max L(a) = \sum\limits_{i = 1}^{n} {(a_{i} - a_{i}^{*} )y_{i} - } \frac{1}{2}\sum\limits_{i,j = 1}^{n} {(a_{j} - a_{j}^{*} )(a_{i} - a_{i}^{*} )K(x_{i} ,x_{j} ) - } \sum\limits_{i = 1}^{n} {(a_{i}^{*} + a_{i} )\varepsilon } } \\ {s.t.\sum\limits_{i = 1}^{n} {( - a_{i}^{*} + a_{i} ) = 0,a_{i} a_{i}^{*} \in [0,C]} } \\ \end{array} } \right. $$

In Eq. (7), $K()$ denotes Lagrange multiplier; $a$ and $a^{*}$ denote Lagrange multipliers. When any one of the Lagrange multipliers is not 0, the variables in the sample become support vectors at this time, and the equation is shown in Eq. (8).

8 $$ K(x_{i} ,x) = e^{{ - \gamma \left\| {x_{i} - x} \right\|^{2} }} $$

In Eq. (8), $\gamma$ denotes the kernel function parameters. Equation (9) depicts the choice function's equation.

9 $$ f(x) = \sum\limits_{i = 1}^{n} {(\alpha_{i} - \alpha_{i}^{*} )e^{{ - \gamma \left\| {x_{i} - x} \right\|^{2} }} + b} $$

**Figure 3:**
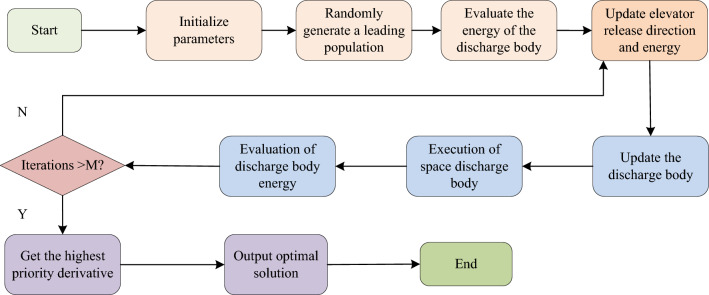
Implementation process of the LSA.

Combining the above descriptions, it can be seen that the accuracy performance, robustness performance and generalisation performance of SVM depend on the insensitivity coefficient and penalty factor of SVM. Therefore, in order to quickly select the optimal parameters of SVM, improve the performance of SVM, and accelerate the training speed of SVM. The study will improve the SVM based on LSA and construct the LSA-SVMPN security risk evaluation algorithm, which is a heuristic search algorithm commonly used in solving combinatorial optimisation problems, with global search capability and high convergence speed.Theoperational steps of the LSA algorithm simulate the behaviour of lightning as it searches for the shortest paths through the cloud layer. It does this by randomly generating a set of solutions, i.e. discharges, in the search space and then updating the discharges according to the fitness of each discharge. The LSA controls the search process based on the location of the current optimal solution and the global ideal solution, while injecting some randomisation to avoid slipping into the local optimal solution.The implementation flow of the LSA is shown in Fig. 3.

As shown in Fig. 3, the discharging body is one of the core concepts of LSA. In the cloud, the discharging body loses kinetic energy when it elastically collides with other particles, and the velocity equation in this process is given by Eq. (10).

10 $$ v_{p} = 1/\sqrt {\left[ {1 - (1/\sqrt {\left( {1 - v_{0} /c} \right)^{2} - sF_{i} /mc^{2} } )^{ - 2} } \right]} $$

In Eq. (10), $v_{0}$ denotes the initial velocity of the discharger; $m$ is the mass of the discharger; $s$ is the motion distance of the discharger after the collision; $c$ is a constant, i.e., the speed of light; and $F_{i}$ is the ionisation rate constant.In the LSA, in addition to the exploration of the target solution space achieved by collision, the discharger can also generate a new branch through the creation of a symmetric path. At this time the new discharge body creation process is shown in Eq. (11).

11 $$ \overline{{p_{i} }} = h + h^{\prime} - p_{i} $$

In Eq. (11), $\overline{{p_{i} }}$ and $p_{i}$ are the two newly generated dischargers, respectively; $h$ and $h^{\prime}$ are the boundary limits of the solution space. In the LSA algorithm, the dischargers are divided into three kinds of dischargers, which are transition dischargers, spatial dischargers and guided dischargers. Among them, the transition discharger can be particleinitialized for LSA, and the spatial discharger is created by using the standard uniformly distributed probability function. And the spatial discharger selects the optimal solution to the space by changing the position of the discharger, which will move the distance to the space. When the maximum number of iterations has been reached andthere are no more optimal solutions, the optimal solution selected at that time becomes the guided discharge body. The equation for the normal probability density function of the lead body is given by Eq. (12).

12 $$ f(x^{L} ) = \frac{1}{{\sigma \sqrt {2\pi } }}e^{{ - (x^{L} - \mu )^{2} /2\sigma^{2} }} $$

In Eq. (12), $x^{L}$ denotes the guided discharge body; $\sigma$ is the scale parameter; $\mu$ is the shape parameter. To avoid slipping into the local optimal solution, it is investigated to introduce a certain degree of randomization in the rows of the LSA, as indicated in Eq. (13).

13 $$ P_{i - new}^{L} = P_{i}^{L} \pm norm(rand(\mu_{L} ,\sigma_{L} )) $$

**Figure 4:**
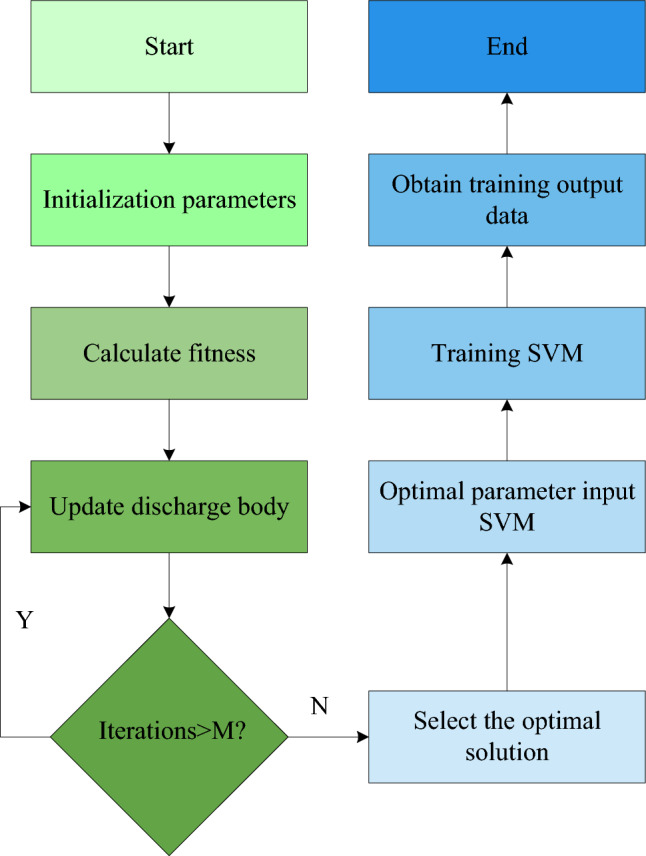
Implementation process of the LSA-SVM algorithm.

In Eq. (13), $rand(\mu_{L} ,\sigma_{L} )$ denotes the random shape parameter and size parameter; $P^{L}$ is the position of the bootstrap discharger; $norm()$ denotes the normal distribution function. In order to construct the LSA-SVMPN security risk assessment algorithm, firstly, the study replaces the parameters of the support vector regression machine with the discharge bodies in the LSA and assigns initial energy to each discharge body. The study then uses LSA to optimise the diffusers to find the optimal diffuser positions. The study then assigns the position coordinates of the discharge bodies to the parameters of the support vector regression machine as parameter values. Finally, the study uses the obtained parameters to train the SVM to assess the PN security risk, resulting in the final LSA-SVMPN security risk assessment method. Figure 4 depicts the implementation flow of the LSA-SVM method developed in the study.

As shown in Fig. 4. The LSA-SVM risk evaluation algorithm constructed in the study is divided into two modules, i.e., the LSA optimal parameter selection module and the SVM security risk assessment module. In the LSA Optimal Parameter Selection module, the study replaces the parameters to be optimised in the SVM with the discharge body in the LSA and initialises the initial parameters of the LSA and SVM. For example, the high-dimensional space weights, the initial energy of the discharge body and the maximum number of iterations. The fitting evaluation phase is then entered. The study evaluates the fitness of all input dischargers. If the initial energy of this discharger is sufficient to complete the exploration of the spatial solution, the symmetric channel is generated and the new space is reached by selecting the channel with higher energy among them. Otherwise, the original energy is used to create a new room discharger and reach the new room. The guided discharger is output as the best option if, when the maximum number of iterations is reached, there is no guided discharger with a higher adaptation value. This is the third and final step. The SVM security risk evaluation stage is started after the optimal parameter selection is completed. After collecting the PN security data, the study takes the network security data as the input training data and preprocesses this data to improve the training efficiency and training speed. The study optimises the penalty factor and insensitive parameters of SVM using LSA in the first module to achieve the ideal parameters. To finish SVM training, the optimal parameters are entered into SVM. Finally, the outcomes of the PN security risk evaluation. The LSA and SVM are effectively integrated and optimized according to the LSA parameter module and the SVM vector mapping. It then provides algorithm guidance for the power grid security risk assessment model.

### PNSREM based on LSA-SVM algorithm

**Figure 5:**
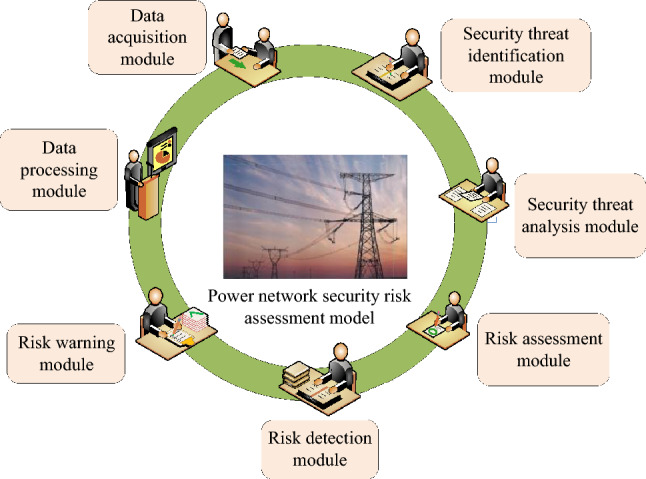
Functional module of the power network security risk assessment model.

PNSREM is a model used to assess the security and risk level of a PCN system. It can assess the security risk of a PN in a quantitative or qualitative manner by collecting, analysing and processing relevant data of the PCN system, and considering various potential security threats and risk factors in a comprehensive manner.The main functional modules of the PN Security Risk Evaluation Model (PNSREM) are shown in Fig. 5.

**Figure 6:**
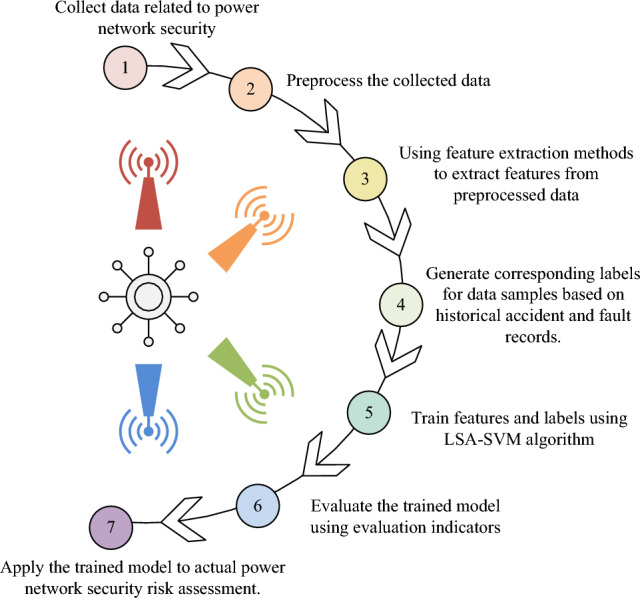
The evaluation process of the power network security risk evaluation model based on LSA-SVM.

A data collection module, a security threat identification module, a security threat analysis module, a risk assessment module, a risk detection module, a risk warning module and a data processing module are the main components of the PN risk security assessment model, as shown in Fig. 5. Among them, the data collection and processing module is mainly responsible for collecting various PCN system data, such as network topology, device status, communication traffic, etc., and processing and preparing the data for further analysis and evaluation. The security threat identification and analysis module, on the other hand, identifies and analyses possible security threats in the PCN system, such as network attacks, malware, information leakage, etc., in order to identify potential security risks. On the other hand, the risk assessment module assesses the security risk of the PN based on the results of the identification and analysis of security threats and the characteristics and requirements of the PN network. This is done using quantitative or qualitative approaches. The result of the assessment is the PN Security Risk Evaluation Index constructed by the study. After completing the analysis and output of PN risks, the risk monitoring and alerting module then conducts real-time monitoring of the security status of the PN to detect abnormal behavior and potential threats to the network in a timely manner and provide early warning to network maintenance personnel so that they can take timely and appropriate security measures and coping strategies to reduce losses due to the impairment of network function. Finally, the PN Security Fengxia Evaluation Module can also store historical data and provide intuitive graphs, reports and other forms in a visual display interface to make it easier for PS managers to understand and analyze the security risk situation of the PCN system. To achieve the above functions, the study integrates the LSA-SVM evaluation algorithm with the PNSREM and constructs the PNSREM based on the LSA-SVM.The evaluation function implementation flow of the model is entered into Fig. 6.

The implementation of the security risk evaluation function is broken down into seven parts, as shown in Fig. 6. The study's first phase is data collecting, which can be accomplished by requesting information from power companies, regulatory bodies, academic institutions, and other sources about PN security. The collected data comprise power system topology, equipment specifications, fault records, historical incident reports, and related information. Following this initial phase, the data undergo preprocessing to streamline calculations, promote efficiency, and minimise errors. This involves essential operations such as data cleaning, noise reduction, and data normalisation. Ensure the accuracy and consistency of the data. The third step is feature extraction of the preprocessed data using LSA-SVM algorithm, the study selects and extracts suitable features to describe the security status and risk factors of PN, such as current, voltage, frequency, load and other related indicators, according to the goal of security risk evaluation.The fourth step is label generation, where the study generates two types of labels, security and risk, for the data samples based on historical accident and failure records in order to train and evaluate the model. The fifth step is the training of the pair of features and labels using the LSA-SVM algorithm. During the training process, LSA-SVM constructs a decision boundary capable of separating normal and fault states based on the features and labels of the training samples. The sixth step is model evaluation and performance optimisation, where the study evaluates the trained model using a test set and calculates the model's accuracy, recall and other metrics on identifying normal and fault states. Based on the evaluation results, the model is optimised and tuned to further improve its evaluation performance. In the final step for using this SREM in real scenarios, it is investigated to classify and evaluate PN security data using the trained ISA-SVM model. Based on the output data from this model, the research may assess the PS's current level of security and potential dangers, and it can also offer support for security management and PS operation. Through the above steps, the IAS-SVM-based PNSREM can achieve the assessment and prediction of PS security risks, and help power operators and decision makers to identify potential security risks in time and take appropriate measures to ensure the safe operation of PSs.

## Empirical analysis of PNSREM based on LSA-SVM algorithm

The project will empirically evaluate both the PNSREM based on this fusion algorithm and the proposed LSA-SVMPN security risk evaluation method to confirm their effectiveness. The study will conduct performance comparison experiments on the LSA-SVM algorithm and performance comparison analysis and satisfaction survey on the PNSREM based on the LSA-SVM algorithm.

### Experimental environment and data preparation

**Figure 7:**
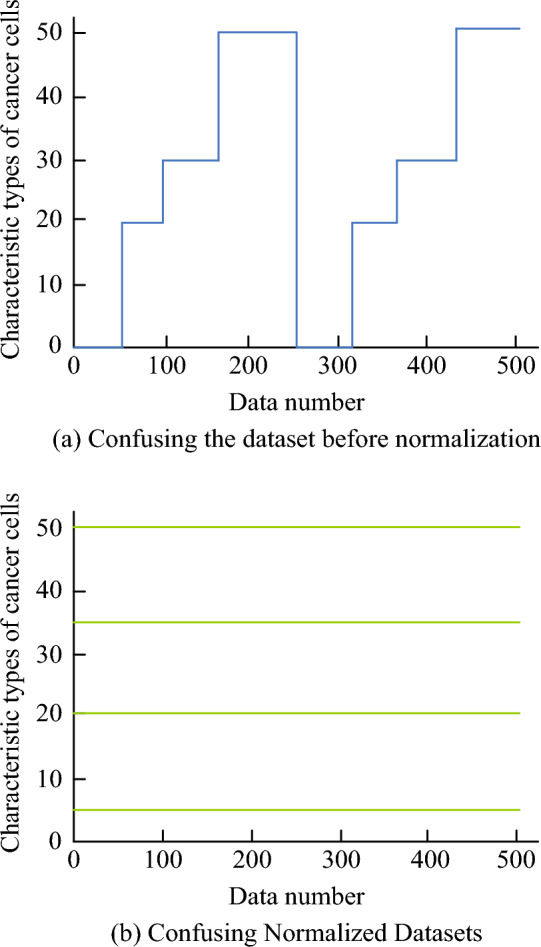
Pre-processed data results.

The study employs LSA to choose the ideal values for the penalty factor and insensitivity coefficients in the SVM, then uses the ideal values to train the SVM to build the LSA-SVMPN security risk evaluation algorithm. The Breast Cancer dataset, which includes 569 samples encompassing 30 attributes, served as the training set for the study. It covers features of breast cancer cells. To expedite the training process, the study applies random normalisation to the data in this dataset. Figure 7 displays the outcomes of the data preprocessing.

**Figure 8:**
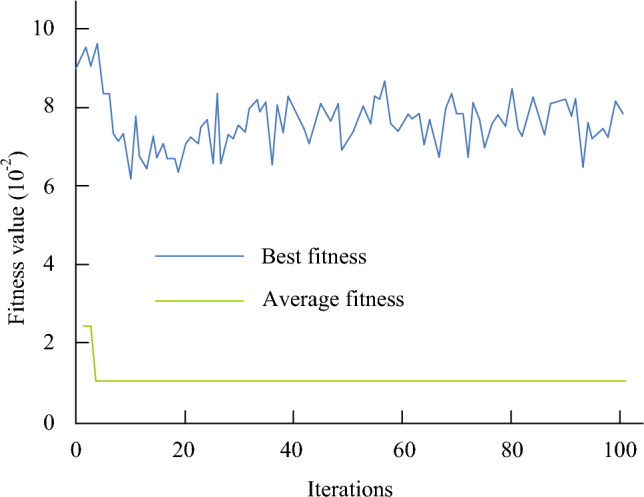
Results of the fitted fitness calculations for the LSA.

The study obfuscates the original data and the normalised data, and the 1600 pre-obfuscation and 400 post-obfuscation data sets are used as training data. The study sets the number of iterations in the LSA to 100 and the initial particles to 50, and performs fitting experiments on it using the test data set to calculate the fitness of the LSA.The results of the fitting fitness calculation of the LSA are shown in Fig. 8.

As illustrated in Fig. 8, the adaptation value of the LSA exhibits significant fluctuations. The highest adaptation value is achieved in 1–5 iterations, during which the LSA selects a penalty factor of 0.1, a kernel function parameter of 0.14, and an insensitivity parameter of 0.001. The optimal parameters selected are then inputted into the SVM algorithm to construct the LSA-SVMPN evaluation model. In order to verify the effectiveness of the LSA-SVMPN security risk evaluation algorithm, the study uses the CIC-IDS 2017 dataset to conduct performance comparison experiments on them respectively. This dataset is a standard dataset published by the Canadian Communications Research Centre for evaluating and studying network intrusion detection algorithms. The dataset covers a number of different attack types such as DoS (Denial of Service), DDoS (Distributed Denial of Service), scanning, malware, etc. The experimental environment for the study was Windows 10 for the operating system; Intel Core i5-1035G1 for the processor model. the live development environment was PyCharm 2019.1.3 and the writing language was Python 3.8. According to the analysis of experimental data set and algorithm parameters, we provide appropriate comparison conditions for the performance test of power grid security risk model, so as to improve the accuracy of experimental data.

### Analysis of the effectiveness of PN security risk evaluation algorithms

**Figure 9:**
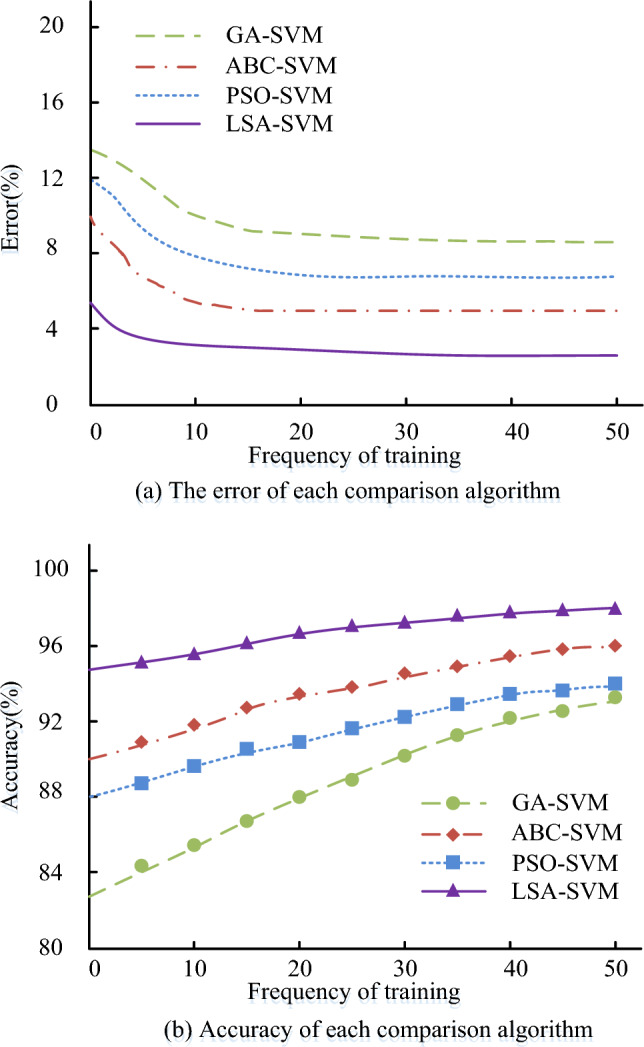
The error and accuracy comparison results of each comparison algorithm.

Through performance comparison experiments using the CIC-IDS 2017 dataset, the study evaluated the effectiveness of the proposed LSA-SVMPN security risk evaluation algorithm. The comparison algorithms are Artificial Bee Colony-Support Vector Machines (ABC-SVM), based on the Artificial Bee Colony algorithm (ABC), Genetic Algorithm-Support Vector Machines (GA-SVM), based on the Particle Swarm Optimization-Support Vector Machines (PSO-SVM) algorithm, and Genetic Algorithm-Support Vector Machines (GA-SVM), based on the Particle Swarm Optimisation algorithm. Accuracy, error, the F1 value, the loss function value, the Receiver Operating Characteristic (ROC) curve, and the Precision Recall Characteristic (ROC) curve are the comparative metrics. Figure 9 displays the accuracy and error comparison results for each comparison algorithm.

**Figure 10:**
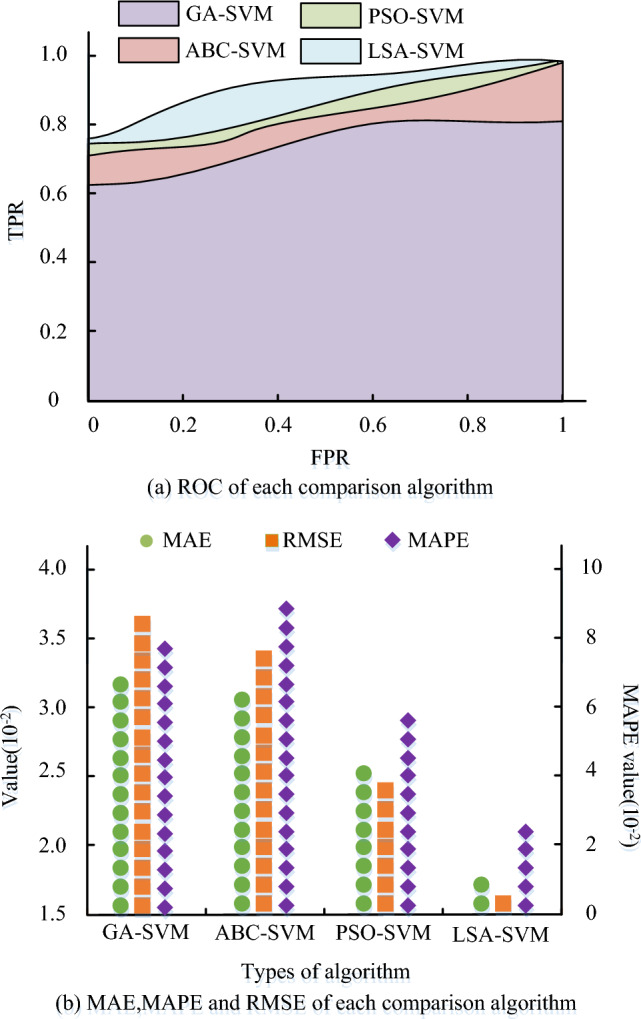
The error and ROC curves for each comparison algorithm.

The error curves for each comparison procedure are displayed in Fig. 9a. According to Fig. 9a, each comparison algorithm's error rate decreases over time until it reaches a stable level. The error rates for the GA-SVM algorithm are 8.1%, ABC-SVM algorithm is 5.2%, PSO-SVM algorithm is 7.4%, and LSA-SVM algorithm is 3.8%. Compared to the other algorithms, the LSA-SVM algorithm has a reduced error rate. The precision percentage of every comparison algorithm is presented in Fig. 9b. In Fig. 10b, the rate stabilizes after reaching a certain count of iterations.The accuracy rates of the GA-SVM algorithm, ABC-SVM algorithm, PSO-SVM algorithm, and LSA-SVM algorithm are all shown, and the accuracy rate of the LSA-SVM algorithm is 96.2%.The study's proposed LSA-SVM method has a lower error than the other comparison algorithms, and it performs more accurately than other algorithms, according to the results, which may be summed up as follows. Figure 10 displays the comparison outcomes for each comparison algorithm for ROC curves, Mean Absolute Error (MAE), Root Mean Square Error (RMSE), and Mean Absolute Percentage Error (MAPE).

**Figure 11:**
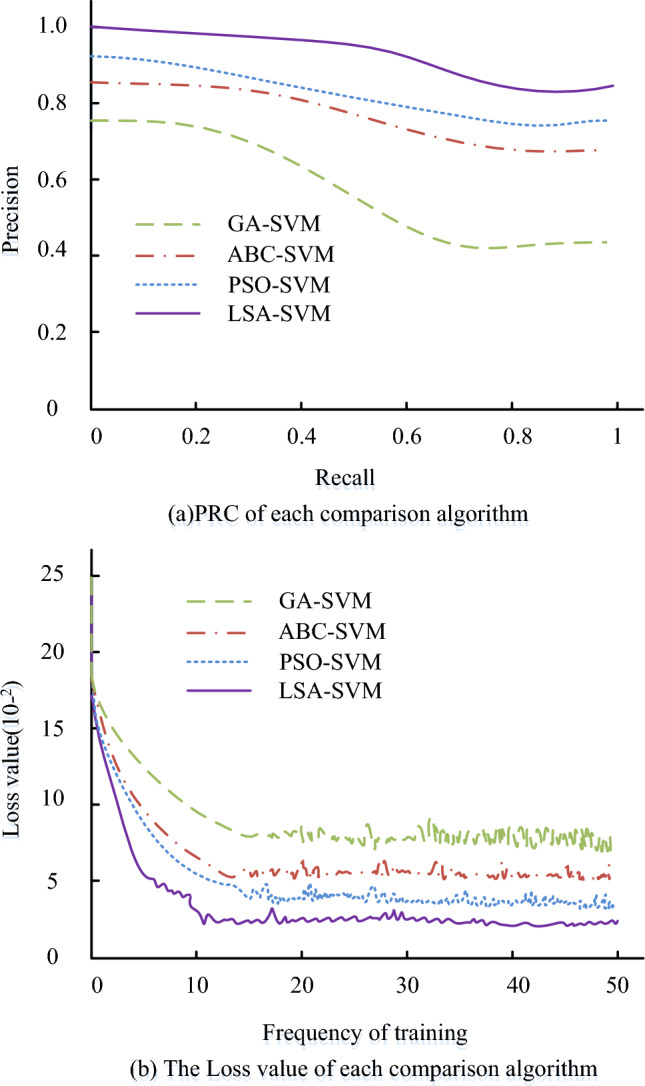
PRC curves and loss function curves of each comparison algorithm.

Figure 10a presents the ROC curves for every comparison algorithm. It is clear that the proposed LSA-SVM algorithm has a higher area under the line of the ROC curve, at 0.93, compared to the other comparison algorithms. These results indicate that the proposed algorithm can effectively distinguish between risky and non-risky data, exhibiting superior accuracy and reliability in risk prediction. The MAE, RMSE, and MAPE values of the comparison methods are displayed in Fig. 10b. Figure 10a demonstrates that the LSA-SVM algorithm outperforms the other comparison algorithms with MAE values of 1.7 × 10^−2^, RMSE values of 1.6 × 10^−2^, and MAPE values of 2.7 × 10^−2^. The suggested LSA-SVM algorithm has better accuracy and dependability than the other algorithms, and has greater potential for use in the field of risk evaluation, according to the results, which are summarised. Figure 11 displays the PRC curves and loss function curves of the comparison algorithms.

The PRC curves for each comparison algorithm are displayed in Fig. 11a. The LSA-SVM algorithm, proposed specifically for this study, outperforms the other algorithms as it is situated closer to the top right corner of the PRC curve. The loss function curve for each comparison algorithm is shown in Fig. 11b, and it can be observed that as the number of iterations increases, the loss function for each comparison method steadily lowers until it tends to be stable. The suggested LSA-SVM algorithm outperforms the previous comparison algorithms in terms of loss function value after stabilisation, loss function convergence speed, and curve fluctuation, with a loss function value of 3.6 × 10^−2^. In summarising the findings, it can be noted that the suggested LSA-SVM algorithm of the study also performs better in recognising high-risk scenarios, which can better assist decision-makers in better anticipating and responding to potential dangers.

### Empirical analysis of PNSREM based on LSA-SVM algorithm

The work uses empirical analysis and expert evaluation to confirm the efficacy of the proposed PNSREM based on the LSA-SVM algorithm. The IEEE-33 node standard test system was chosen for the example test, and relevant data, such as node, line, and load data, were collected from the IEEE benchmark test system. Therefore, the power grid data was systematically tested, and the importance of the first five serial numbers of IEEE-33 node was compared with the other indicators to verify the validity of the model. The details are shown in Table 2.

**Table 2:** Sorting results of IEEE-33 nodes on different indicators.

Serial number	LSA-SVM algorithm		Tightness centrality	Electric betweenness	PageRank
	Importance value	Node	Node	Node	Node
1	1.003	6	6	6	10
2	0.632	10	10	10	6
3	0.589	12	4	4	12
4	0.563	27	12	9	27
5	0.486	4	28	12	15

**Figure 12:**
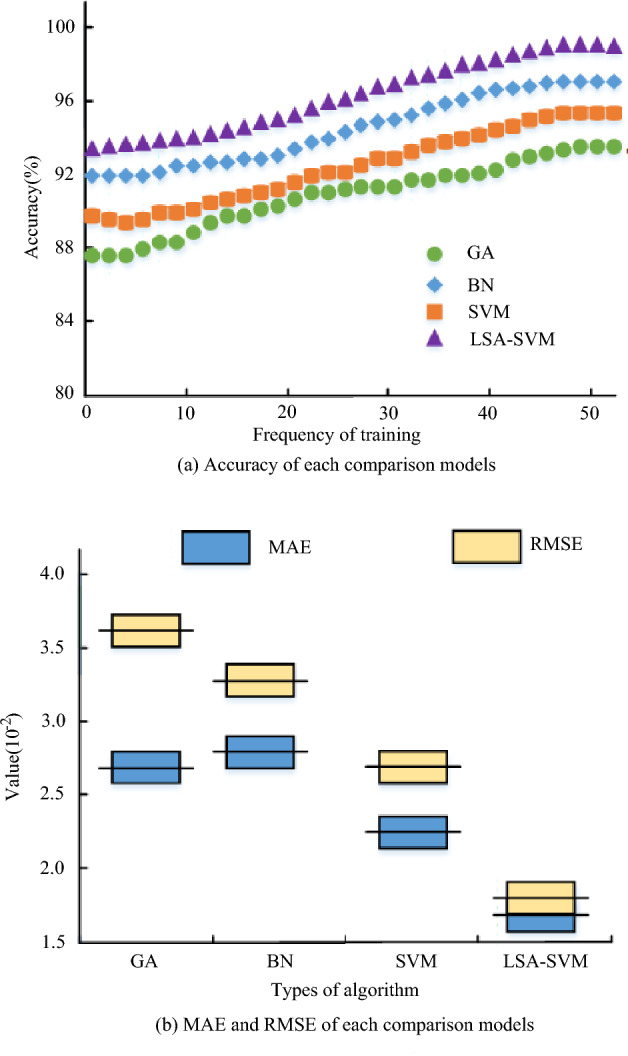
Accuracy and error comparison results of each model.

From Table 2, the identification of the key points of the power grid is different according to different methods. In the importance ranking of IEEE-33 nodes, most serial numbers have 8 in the top 10 indexes of closeness centrality and PageRank, and the ranking of node 12 shows that it is robust to power grid structure and transmission efficiency. Therefore, the LSA-SVM algorithm has an excellent discrimination effect on the power grid data. To ensure the accuracy and completeness of the data, meticulous measures were taken.And the comparison models included the GA-based PN security risk assessment model, the Bayesian network (BN)-based PN security risk assessment model, and the conventional SVM-based PN security risk assessment model. The performance evaluation metrics are accuracy, error, computation time, and expert score. The comparison results of accuracy, MAE and RMSE of each comparison model are shown in Fig. 12.

**Figure 13:**
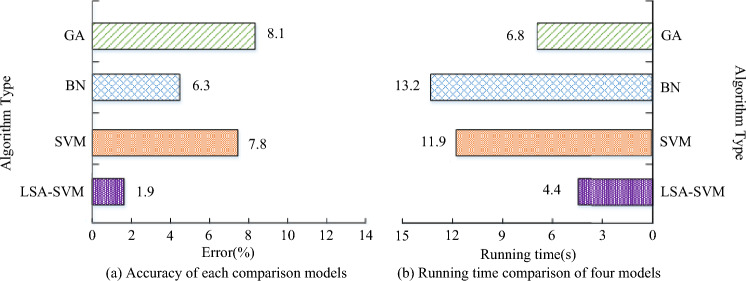
Comparison results of the error rate and operation time of each risk evaluation model.

The accuracy curve of each comparison model is shown in Fig. 12a, and it can be observed that as the iteration coefficients grow, so does the evaluation accuracy of each risk evaluation model until it tends to be stable. The accuracy curve of the LSA-SVM risk evaluation model suggested in the study is higher than that of the other comparison models, and its accuracy after stabilisation is 97.8%, making it more accurate than the other comparison models in terms of evaluation accuracy performance. Figure 12b compares the MAE and RMSE results for each comparison model. In Fig. 12b, the proposed LSA-SVM risk assessment model in this study had significantly lower MAE and RMSE values than the other comparison models, with MAE values of 1.64 × 10^−2^ and RMSE values of 1.78 × 10^−2^. Taken together, the above results show that the research-proposed LSA-SVM risk evaluation model has better evaluation accuracy performance than other comparative models and has practical application value. The comparison results of the error rate and calculation time of each risk evaluation model are shown in Fig. 13.

**Figure 14:**
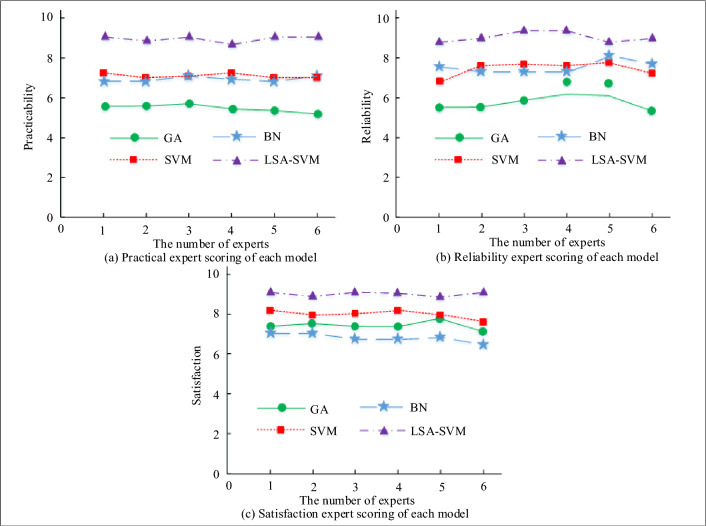
Expert scoring results.

The error rate for each comparison model is shown in Fig. 13a, and it can be seen that the GA risk evaluation model has an error rate of 8.1%, the BN risk evaluation model has an error rate of 6.3%, the GA risk evaluation model has an error rate of 7.8%, and the LSA-SVM risk evaluation model has an error rate of 1.9%. This means that the LSA-SVM risk evaluation model has a lower evaluation error than the other comparison models. Meanwhile, the suggested LSA-SVM risk assessment approach is better equipped to deal with the present network risk scenario when compared to the existing literature^10^. According to Fig. 13b, which compares the computing times of each comparison model, the LSA-SVM risk evaluation model's computing time is lower than that of the other comparison models (GA risk evaluation model computing time is 6.8 s, BN risk evaluation model computing time is 13.2 s, SVM risk evaluation model computing time is 11.9 s, and LSA-SVM risk evaluation model computing time is 4.4 s). Although the random search method of GA has good applications in combinatorial optimization and global optimization of initial populations, and is suitable for various general problems, the GA draws on the evolutionary laws of the biological world, and cannot fully grasp the randomness of power grid risk factors. BN mainly solve the problems of uncertainty and incompleteness in digital models, using node relationships and conditional probabilities to effectively control influencing factors. However, the evaluation factors of power grid security risks usually do not rely on the decision-making of control factors, so the evaluation effect of power grid security risks is not significant. The SVM algorithm is feasible in the application of pattern recognition, but it is easily affected by the sample parameters of geometric decision boundaries, making the classifier's results meet the minimum optimization criteria. In the face of complex power grid safety risk assessment models, the parameters and sample tests considered are relatively rich. Therefore, the study uses LSA algorithm to improve and enhance SVM algorithm, which not only improves the accuracy of model classification and recognition, but also enriches the testing frequency of samples, thereby satisfying the operational performance of power grid safety risk assessment models. The study used the expert assessment approach to rate the applicability, dependability, and satisfaction of each model in order to further assess the quality and worth of LSA-SVMPNSREM. The expert scoring results are shown in Fig. 14.

In Fig. 14a, the practical expert score of the LSA-SVM risk evaluation model is between 8 and 10, which is the highest score among the four models. Figure 14b shows the reliability evaluation results of each model, and from Fig. 14b, it can be seen that the reliability score of the LSA-SVM risk evaluation model proposed in the study is 8.9, which is higher than the other comparison models. Figure 14c illustrates the results of the satisfaction evaluation for each model. The satisfaction score for the LSA-SVM risk evaluation model proposed by the research is 9.3. In addition, the performance of the LSA-SVM risk assessment model was compared with the other algorithms or models, and the results are shown in Table 3.

**Table 3:** Performance comparison of power grid safety risk assessment.

Methods	Accuracy (%)	Error rate (%)	Time efficiency
Cloud models and a random forest	95.26	4.74	1.06 s
Improve the grey Wolf algorithm	93.57	6.43	3.15 s
The niche genetic algorithm	87.64	12.36	4.67 s
Subliminal model	88.52	11.44	4.02 s
LSA-SVM model	98.61	1.39	0.87 s

**Figure 15:**
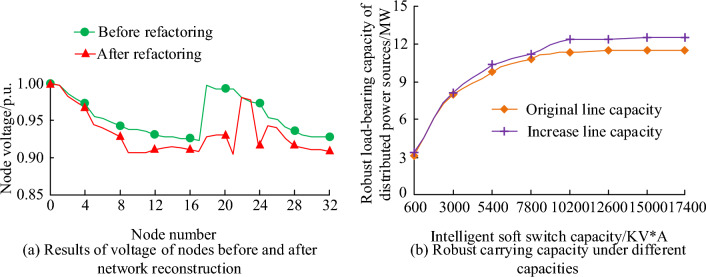
Evaluation results of network reconstruction and intelligent soft switching.

From Table 3, the LSA-SVM risk assessment model has a high accuracy of 98.61% and an error rate of 1.39%, which is the better performance of the five methods. In addition, the cloud model and random forest methods are similar to the LSA-SVM model, with 1.06 s and 0.87 s, respectively. Therefore, it proves the superior performance of LSA-SVM risk assessment model, which has fast prediction judgment and good assessment results in the protection of power grid security. The LSA-SVM risk assessment model benefits from the integration of LSA and SVM, substituting the discharge body in the LSA instead of the parameters of the SVM and assigning initial energy to each discharge. LSA to optimize the diffuser to find the optimal diffuser location. Finally, the parameters of the position coordinates assigned to the SVM are used as the parameter values. Therefore, LSA trained the safety risk assessment of SVM on the parameters of PN to obtain the LSA-SVMPN. In addition, for the robust carrying capacity of LSA-SVM model in the distribution network, the power grid adopts network reconstruction and intelligent soft switch to improve its robust carrying capacity. Therefore, the network reconstruction and the intelligent soft switch are accurately evaluated, and then the robustness and generalization ability of the model are expounded. The model was evaluated using the IEEE-33 node distribution network, as shown in Fig. 15.

It is obtained from Fig. 15 that the voltage adjustment of network reconstruction can affect the robust carrying capacity of the model, and the capacity adjustment of intelligent soft switch also has a promoting effect on the robust carrying capacity of the distribution network. In Fig. 15a, it is concluded that the voltage of each IEEE-33 node decreases after network reconstruction, and finally basically remains at around 0.92p.U. In Fig. 15b, it is concluded that with the increasing capacity of the intelligent soft switch, the robust capacity of the distributed power supply is also increasing until it remains at 13 MW. A 2 MW increase compared to the original line. Therefore, it is proved that the LSA-SVM model has good robustness and generalization ability to the node voltage and intelligent soft switching capacity of the distribution network.

**Figure 16:**
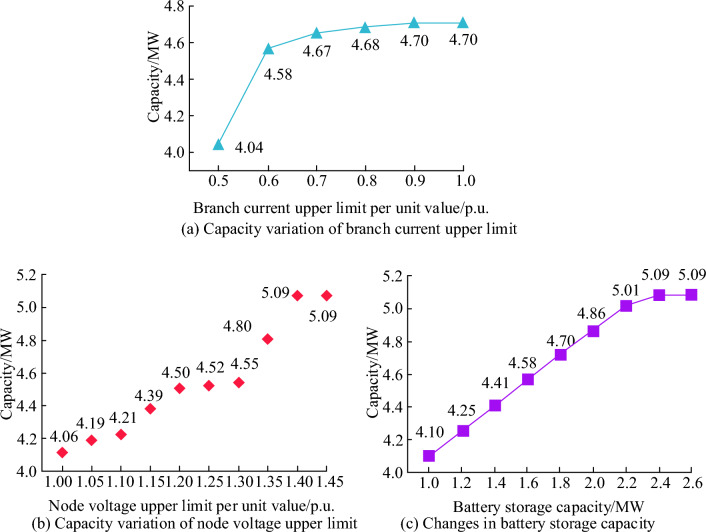
Comparison of distributed photovoltaic carrying capacity of AC/DC hybrid distribution networks under different production parameters.

Finally, based on the quantitative analysis and qualitative evaluation methods of power grid risk assessment, the LSA-SVM model was used to perform static evaluation analysis on the algorithm operation and accuracy of safety risk indicators. To demonstrate the robustness and generalization ability of LSA-SVM models in different scenarios, safety constraint testing was conducted on distributed photovoltaic access nodes in AC/DC hybrid distribution networks. Compare the carrying capacity of the branch current upper limit, node voltage upper limit, and battery energy storage capacity. As shown in Fig. 16.

From Fig. 16, the unit values of the upper limit of branch current and node voltage, as well as the saturation point values of the battery storage capacity during the increase process, are 0.8p.u, 1.4p.u, and 2.4 MW, respectively. In Fig. 16a, it is shown that when the upper limit of branch current per unit value is 0.8p.u, the maximum carrying capacity of distributed photovoltaics in the AC/DC hybrid distribution network gradually increases, which is 4.7 MW. As the upper limit of node voltage per unit value increases in Fig. 16b, the carrying capacity of its distributed photovoltaic system continues to increase, with a maximum capacity of 5.09 MW. In Fig. 16c, the relationship between battery storage capacity and the carrying capacity of distributed photovoltaics is shown. When the battery storage capacity is 2.4 MW, the carrying capacity of the distribution network is 5.09 MW. Therefore, it indicates that the energy storage capacity of the hybrid AC/DC distribution network has been improved, which further demonstrates the superiority of the LSA-SVM model in terms of robustness and generalization ability.

However, considering the evaluation of investment quotations for power grid engineering, economic benefits are included in the evaluation indicators and dynamically evaluated and analyzed to provide comprehensive data for safety measures and emergency plans of power grid engineering. Therefore, based on the quotation of relevant equipment in the distribution network, the LSA-SVM model can accurately identify and defend the safety data of the power grid. This prolongs the life cycle of power grid engineering equipment and reduces the loss of investment income, thereby reducing the cumulative engineering quotation of power grid operating equipment. Therefore, the LSA-SVM model provides an efficient and accurate measure of the likelihood of security risks for dynamic and complex power grid security risks, while also facilitating the identification and analysis of risk assessment modules and the formulation of measures.In summary, the practicality, reliability and satisfaction scores of the research-proposed LSA-SVM risk evaluation model are higher than those of the other comparative models, and it has a great potential for application.

## Conclusion

The society is becoming more and more concerned with PN security as a result of the rapid advancement of communication technologies. The research builds the LSA-SVMPN security risk evaluation algorithm by integrating the LSA algorithm with the SVM algorithm and then builds the LSA-SVMPNSREM on top of it in order to increase the accuracy and processing speed of the conventional PNSREM. The accuracy of the proposed LSA-SVM algorithm is 96.2%, which is higher than the other comparative algorithms; the error rate of the LSA-SVM algorithm is 3.8%; the MAE value of the LSA-SVM algorithm is 1.7 × 10^−2^; the RMSE value of the LSA-SVM algorithm is 1.6 × 10^−2^; the MAPE value of the LSA-SVM algorithm is 2.7 × 10^−2^; and the MAPE value is lower than the other comparative algorithms whereas the loss function value of the LSA-SVM algorithm is 3.6 × 10^−2^, and the convergence speed is faster than the other algorithms, which is lower than the other comparison algorithms. The accuracy of the LSA-SVM risk evaluation model is 97.8%, higher than that of other comparative models. Its MAE value is 1.64 × 10^−2^, its RMSE value is 1.78 × 10^−2^, and its error rate is 1.9%, lower than that of other comparative models. Its operation time is 4.4 s, shorter than that of other comparative models. Its practicality is also higher than that of other comparative models. In conclusion, the LSA-SVM algorithm and the PNSREM based on the LSA-SVM algorithm proposed by the study perform more accurately and quickly than other methods, and they have a wide range of potential applications. They can be used as auxiliary support for enhancing the security and reliability of PS. The study will further optimise the generalisation ability of LSA-SVMPNSREM and simultaneously verify its efficacy in realistic application scenarios.

The LSA-SVM model efficiently integrates lightning search technology and support vector machine. It first simulates the shortest path of lightning search through clouds, and randomly generates a set of solutions, that is, discharge, in the search space. Secondly, the discharge is updated according to the fitness of each discharge, and the position of the global ideal solution, while injecting some randomization is used to avoid sliding into the local optimal solution. Finally, the operation method of SVM is used to transform the data of low-dimensional space into high-dimensional space through vector mapping, thus increasing the stability and encryption of data security. Thus demonstrating the robustness of the LSA-SVM model to the power grid system.

With the advancement of technology and the development of power grid systems, complex systems are constantly being constructed in terms of operation mechanisms, intelligent devices, emergency management, and energy integration. The LSA-SVM model is susceptible to the influence of evaluation indicators and their weights on the operational efficiency and risk assessment of complex power networks, which can lead to distorted results in its risk assessment algorithm. In addition, the LSA-SVM model lacks consideration of multiple factors and experimental analysis for risk assessment in more complex and systematic power grid network environments. Therefore, further exploration and analysis of the influencing factors of the complex environment of the power grid are needed in future research. At the same time, in practical applications, the safety protection and stable operation of the power system will face more complex challenges in the future.

## Data Availability

In the current study, the Breast Cancer dataset is available at the "https://www.zybuluo.com/spiritnotes/note/295894" link in the Sklearn database. The CIC-IDS 2017 dataset is available on the Kaggle official website of the "https://www.kaggle.com/datasets/chethuhn/network-intrusion-dataset" Link to get.
